# Computational Identification of Protein Pupylation Sites by Using Profile-Based Composition of *k*-Spaced Amino Acid Pairs

**DOI:** 10.1371/journal.pone.0129635

**Published:** 2015-06-16

**Authors:** Md. Mehedi Hasan, Yuan Zhou, Xiaotian Lu, Jinyan Li, Jiangning Song, Ziding Zhang

**Affiliations:** 1 State Key Laboratory of Agrobiotechnology, College of Biological Sciences, China Agricultural University, Beijing, 100193, China; 2 Advanced Analytics Institute and Centre for Health Technologies, University of Technology, Sydney, 81 Broadway, NSW 2007, Australia; 3 National Engineering Laboratory for Industrial Enzymes and Key Laboratory of Systems Microbial Biotechnology, Tianjin Institute of Industrial Biotechnology, Chinese Academy of Sciences, Tianjin, 300308, China; 4 Monash Bioinformatics Platform and Department of Biochemistry and Molecular Biology, Faculty of Medicine, Monash University, Melbourne, VIC 3800, Australia; Huazhong University of Science and Technology, CHINA

## Abstract

Prokaryotic proteins are regulated by pupylation, a type of post-translational modification that contributes to cellular function in bacterial organisms. In pupylation process, the prokaryotic ubiquitin-like protein (Pup) tagging is functionally analogous to ubiquitination in order to tag target proteins for proteasomal degradation. To date, several experimental methods have been developed to identify pupylated proteins and their pupylation sites, but these experimental methods are generally laborious and costly. Therefore, computational methods that can accurately predict potential pupylation sites based on protein sequence information are highly desirable. In this paper, a novel predictor termed as pbPUP has been developed for accurate prediction of pupylation sites. In particular, a sophisticated sequence encoding scheme [i.e. the profile-based composition of *k*-spaced amino acid pairs (pbCKSAAP)] is used to represent the sequence patterns and evolutionary information of the sequence fragments surrounding pupylation sites. Then, a Support Vector Machine (SVM) classifier is trained using the pbCKSAAP encoding scheme. The final pbPUP predictor achieves an AUC value of 0.849 in10-fold cross-validation tests and outperforms other existing predictors on a comprehensive independent test dataset. The proposed method is anticipated to be a helpful computational resource for the prediction of pupylation sites. The web server and curated datasets in this study are freely available at http://protein.cau.edu.cn/pbPUP/.

## Introduction

The bacterial prokaryotic ubiquitin-like protein (Pup) is initially perceived as a small protein related to post-translational modifications (PTMs). Pup is an intrinsically unstructured protein consisting of 64 amino acids [1, 2]. In the tagging system referred as pupylation, this protein covalently attaches to target lysines for proteasomal degradation by forming isopeptide bonds [3–5]. In eukaryotes, the ubiquitin-proteasome degradation pathway was discovered in the late 1970's [6], while the analogous Pup-proteasome pathway was not identified in prokaryotes until recently [5, 7, 8]. To date, the proteasomal Pup has been discovered in the phyla Actinobacteria and Nitrospira species [9]. The evidence of Pup proteasome degradation pathway has been rapidly accumulating in both the *in vitro* [10, 11] and *in vivo* systems [12].

Pupylation and ubiquitylation are functionally identical but their enzymology is different. In general, ubiquitylation requires three types of enzymes: ubiquitin-activating enzymes, ubiquitin-conjugating enzymes, and ubiquitin ligases [13]. Comparatively, the pupylation process involves two enzymes: one is the deamidase of Pup (DOP) which deamidates the C-terminal glutamine of Pup to glutamate [14, 15], and the other is the proteasome accessory factor A (PafA) which proceeds the deamidase process by attaching Pup to a specific lysine [16, 17]. More specifically, pupylation enzymes are originated from bacterial organisms and show no homology to ubiquitylation enzymes [18, 19].

The Pup-proteasome degradation pathway plays a nutritional role under nitrogen starvation by recycling amino acids [20]. This proteasomal pathway is also critical for the virulence of bacteria [21, 22]. Therefore, identification of pupylated substrates is fundamentally important for understanding both the physiological and pathological mechanisms. A number of large-scale proteomic studies have been performed to identify pupylated proteins based on the molecular signature of pupylated sites [23–27]. Despite the increasing number of experimentally determined pupylated proteins, the underlying mechanism of protein pupylation specificity remains largely unknown [25]. On the other hand, large-scale experimental identification of pupylation substrates is laborious, time-consuming and costly. As an alternative, accurate and cost-effective prediction methods can be used to complement the experimental efforts.

Up to now, a few computational approaches have been developed to predict pupylation sites [28–31]. Xue and co-workers [30] proposed the first predictor named GPS-PUP, which was developed from their original Group-based Prediction System (GPS) with three procedures (i.e. weight training, motif length selection, and matrix mutation) for performance improvement. In 2013, Tung [29] used a training dataset collected from the PupDB database [32] and an encoding scheme called the composition of *k*-spaced amino acid pair (CKSAAP) to develop a predictor called iPUP. Support Vector Machine (SVM) together with a backward feature selection method was used to train the classifier. Both GPS-PUP [30] and iPUP [29] predictors yielded good performance for predicting pupylation sites. In particular, they achieved higher specificity, although their sensitivity was generally low. More recently, Chen et al. [31] developed a predictor PupPred based on balanced training data (1:1 ratio of positive to negative samples). To train the classifier, PupPred combined the k-nearest neighbor (KNN) algorithm with a variety of features including binary features, amino acid pairs, protein secondary structures, position-specific scoring matrix (PSSM) and physicochemical properties. They demonstrated that the encoding of amino acid pairs, the implementation of F-measures for feature selection and the SVM-based classifier contributed to the improved performance of PupPred.

However, the overall performance of the aforementioned three existing predictors is still not satisfying and there is enough room for improvement. To develop a machine learning-based predictor, it is important to devise an appropriate encoding scheme to represent the sequence fragments surrounding pupylation/non-pupylation sites. In the current study, we develop a new SVM predictor named pbPUP based on an improved CKSAAP encoding, i.e. the profile-based composition of *k*-spaced amino acid pairs (pbCKSAAP). The traditional CKSAAP encoding has been widely and successfully used in diverse bioinformatics prediction tasks, such as the prediction of pupylation sites [29], flexible/rigid region [33], O-glycosylation sites [34], ubiquitination sites [35], palmitoylation sites [36], methylation sites [37] and phosphorylation sites [38]. Compared with the traditional CKSAAP encoding, the pbCKSAAP encoding scheme has the advantage of integrating the sequence evolutionary information from the profile (i.e. PSSM) generated by PSI-BLAST search. Originally developed for the prediction of membrane protein [39], pbCKSAAP has revealed more powerful performance in some applications such as the prediction of bacterial pathogen effectors [40].

In this study, the pbPUP predictor was constructed using the training dataset of iPUP [29]. An independent test dataset [25, 29] was used for making fair performance comparison among different methods. The results indicated that pbPUP achieved significantly improved performance on the independent tests compared with other existing methods. Moreover, we also conducted a series of computational analyses to provide in-depth understandings of the pbCKSAAP encoding. Finally, the proposed method pbPUP has been implemented as a web server. Taken together, the current study provides a useful tool for predicting pupylation sites as well as valuable insights into the important sequence patterns surrounding pupylation sites.

## Materials and Methods

**Fig 1:**
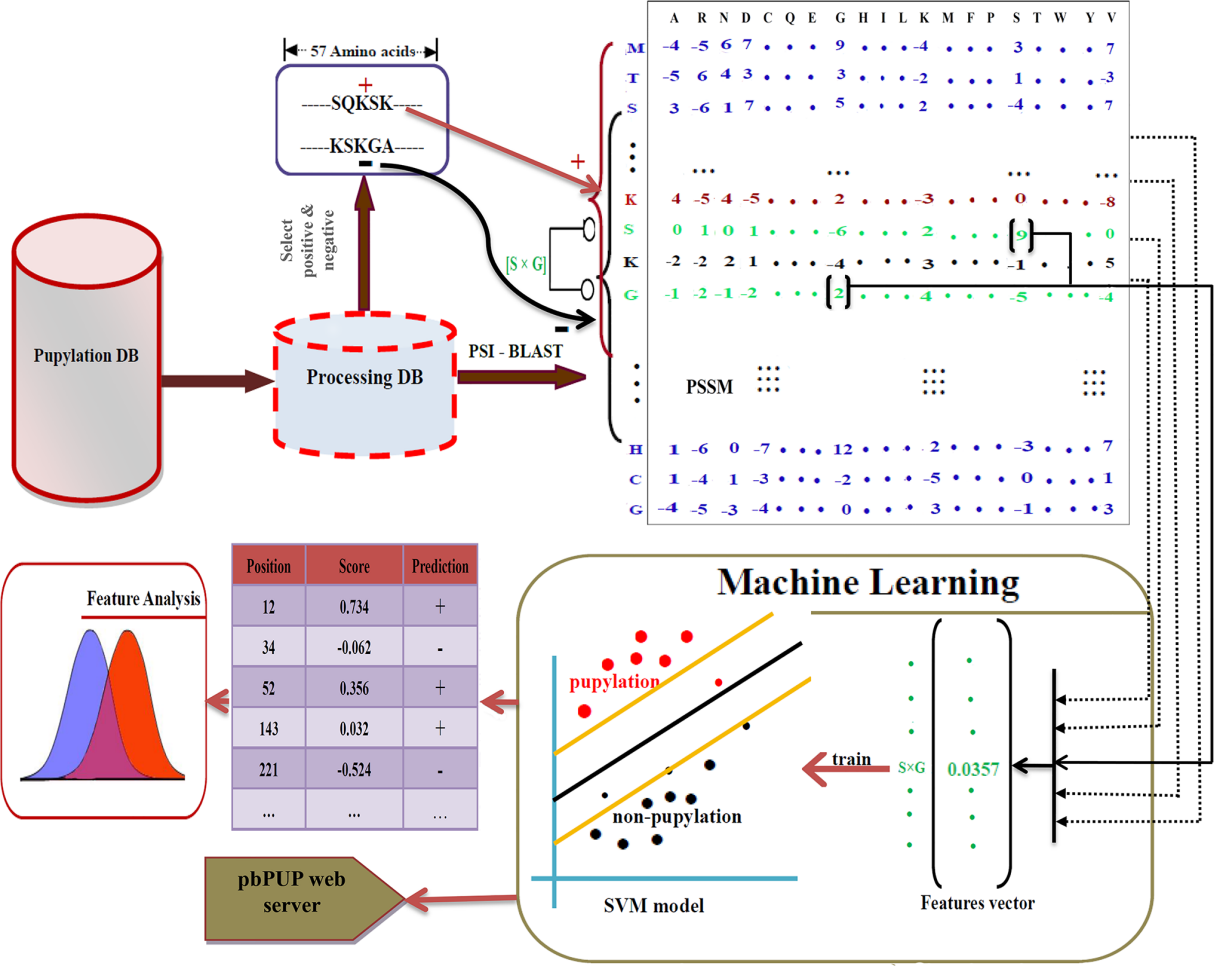
Overview of the proposed pbPUP predictor. The full-length sequence of a pupylated protein is first used to generate the PSSM profile by running PSI-BLAST search against the NCBI NR90 database. Meanwhile, the PSSM matrixes corresponding to pupylation and non-pupylation sites are extracted from the whole profile. The encoded profile-based features are used as the input to train a SVM classifier. After optimization of the SVM parameters, the best SVM model is constructed based on the 10-fold cross-validation performance. Finally, a web server pbPUP is implemented and made available for interested users to predict the potential pupylation sites from the submitted proteins.

In brief, pbPUP is an SVM-based predictor, which was constructed using the pbCKSAAP sequence encoding scheme. An overview of the computational framework of the proposed pbPUP predictor is shown in Fig 1.

### Data preparation

In this study, two datasets were used to train and assess the proposed pbPUP predictor, including the training dataset of iPUP (S1 Table) and an independent test dataset (S2 Table). The experimentally validated pupylation sites (lysine residues) were considered as positive samples, while all the remaining lysine residues that have not been verified as pupylation sites in these proteins were considered as negative samples (i.e. non-pupylation sites). Each site was represented as a sequence fragment with lysine (K) in the center. These two datasets are also summarized in Table 1.

**Table 1:** The statistics of pupylated proteins and their pupylation sites used in this study.

	The iPUP training dataset	Independent test dataset
Number of pupylated proteins	162	71
Number of pupylation lysines	183	86
Number of non-pupylation lysines	2205 (366)	1136

Values in parentheses represent the number of sites used in this study.

The iPUP training dataset was previously compiled to train the iPUP predictor [29], which includes 162 pupylated proteins covering 183 positive and 2205 negative sites. The iPUP training dataset was also employed to train our pbPUP predictor. The numbers of positive and putative negative samples are highly imbalanced in the original iPUP training dataset (~1:12); this imbalance will hamper model training. Therefore, a relatively balanced dataset with a 1:2 ratio of positives to negatives (i.e. 183 positive sites and 366 randomly selected negative sites) was compiled to train our pbPUP predictor.

An independent test dataset was also compiled to benchmark the prediction performance of different predictors. First, 20 pupylated proteins, originally used as the independent test data of iPUP, was directly used in our work. Moreover, we also collected 55 pupylated proteins from a recent work [25]. Among these 55 proteins, the lysine positions of four proteins did not match with the UniProt database (http://www.uniprot.org/). Thus, these four proteins were removed from our study. Finally, we obtained an independent dataset containing 71 proteins with 86 experimentally validated pupylation sites and 1136 putative non-pupylation sites. In the independent test, all the pupylation and non-pupylation sites were used to assess the performance of different predictors. We believe that the performance assessed using the highly imbalanced data could reflect the real applications of different predictors.

### Encoding scheme of pbCKSAAP

The encoding scheme of pbCKSAAP has been used in previous studies [39, 40]. Briefly, a *k*-spaced amino acid pairs can be represented as *p*_*i*_ *{k}p*_*j*_(*i*, *j* = 1, 2, …, 20), where *p*_*i*_ and *p*_*j*_ denote any two residues of the 20 amino acid types. When *k* = 0, *p*_*i*_ *{k}p*_*j*_ stands for a dipeptide and a total of 20×20 = 400 different dipeptides should be taken into account. In this work, *k* = 0, 1, 2, 3 and 4 were jointly considered (i.e. *k*_*max*_ = 4). Thus, the feature vector of each pupylation/non-pupylation site has a dimensionality of 400×5 = 2000. To conduct the pbCKSAAP encoding, each protein sequence was searched by PSI-BLAST against the NCBI NR90 database (version of December 2010) to generate a profile (i.e. PSSM matrix). The e-value cutoff for the inclusion of new sequences and iteration times were set as 1.0×10^−4^ and 3, respectively. For each pupylation/non-pupylation site, the corresponding PSSM matrix was extracted from the whole profile. If an amino acid pair *p*_*i*_ *{k}p*_*j*_ appears between the residue positions *t* and *t*+*k*+1 in the PSSM matrix, the composition score can be calculated using the following equation:

$$S_{i,j}=\sum_{}^{\text{N}}\max\{\min\{\text{PSSM}(t,p_{i}),\text{PSSM}(t+k+1,p_{j})\},0\}$$ (1)

where PSSM (*t*, *p*_*i*_) denotes the score of amino acid *p*_*i*_ at the *t* ^th^ row position of PSSM, PSSM (*t*+*k*+1, *p*_*j*_) stands for the score of amino acid *p*_*j*_ at the (*t*+*k*+1)^th^row position of PSSM, N means *p*_*i*_ *{k}p*_*j*_ appears N times in the pupylation/non-pupylation site. Furthermore, we normalized *S*_*i*,*j*_ using the following formula:

$$S_{i,j}^{\prime }=\frac{S_{i,j}}{L-k-1}$$ (2)

where *L* denotes the total length of sequence fragment, i.e. window size = *L*. Finally, we generated the pbCKSAAP encoding, which is a 2000-dimensional feature vector for each pupylation/non-pupylation site.

To investigate the evolutionary conservation of pupylated or non-pupylated sites, we calculated the average PSSM value (APV) of each position (i.e. the average of each row of the PSSM matrix) in the flanking sequence fragments of each pupylated/non-pupylated site. These APVs were further averaged. More specifically, because the optimal window size in this study was 57, the APVs of the positions [–28,–1] were averaged to obtain the APV of the upstream sites, while the APVs of the positions [+1,+28] were averaged to obtain the APV of the downstream sites.

### Encoding scheme of CKSAAP

Compared with pbCKSAAP, the encoding scheme of CKSAAP is quite simple, which can be directly calculated from the sequence fragments of pupylation/non-pupylation sites. By effectively representing the short sequence motif information in protein sequences or fragments, CKSAAP is an important encoding scheme in many prediction tasks [29, 34–36, 38, 39]. In this work, we retrained the SVM model using the CKSAAP encoding scheme with the purpose of comparing the performance between pbCKSAAP and CKSAAP. To conduct a stringent comparison, the same window size and the same *k*_max_ value were adopted. Thus, a 2000-dimensional feature vector was also generated in the CKSAAP encoding scheme. More details about the CKSAAP encoding can be found in our previous studies [34, 35].

### Feature selection

For a pupylation site, the proposed pbCKSAAP encoding represents its flanking sequence pattern in a comprehensive manner, resulting in a high-dimensional, partially redundant feature vector. It is well known that there could be some key residues or motifs which contribute significantly to the identification of PTM sites [34, 41, 42]. However, it would be challenging to readout the key residues or motifs directly from the high-dimensional feature vector of the pbCKSAAP encoding. Therefore, we employed a well-established dimensionality reduction method, Chi-Squared (*χ* ^2^) to characterize the top ranking features [39]. Let *X* be a feature with *n* possible values *x*_1_, *x*_2_, …, *x*_*n*_ with the probability *P*(*X* = *x*_*j*_) = *p*_*j*_. Then, for a dataset with *c*_*tot*_ positive samples and *d*_*tot*_ negative samples, the *χ* ^2^ score of this feature can be calculated using the following formula:

$$\chi^{2}=\sum_{j=1}^{n}\left[\frac{{\left(c_{j}-c_{tot}\cdot p_{j}\right)}^{2}}{c_{tot}\cdot p_{j}}+\frac{{\left(d_{j}-d_{tot}\cdot p_{j}\right)}^{2}}{d_{tot}\cdot p_{j}}\right]$$ (3)

In addition to the aforementioned variables (*p*_*j*_, *c*_*tot*,_ *d*_*tot*_), *c*_*j*_ is the observed numbers of the positive samples whose feature value *X* = *x*_*j*_, while *d*_*j*_ is the observed numbers of the positive samples whose feature value *X* = *x*_*j*_. By definition, a larger value of *χ* ^*2*^ indicates that the corresponding feature has a greater impact on the discrimination capability of the predictor. More information about the *χ* ^*2*^ feature selection method can be found in the literature [39].

### Model training

In our study, SVM was used to build the classifiers to distinguish the pupylation sites from non-pupylation sites. As an efficient machine learning algorithm, SVM has been widely used in protein bioinformatics [43–48]. In this work, the LIBSVM package (http://www.csie.ntu.edu.tw/~cjlin/libsvm/) was used as an implementation of SVM to train the classifiers [49]. The kernel radial basis function (RBF) was selected and two parameters *C* and *γ* were optimized based on the training dataset through a grid search provided by the LIBSVM package. The ranges of both *C* and *γ* were set as [2^−7^, 2^8^], which resulted in 225 grids. All the grids were evaluated based on 10-fold cross validation in order to find the optimal parameter combination of *C* and *γ*.

### Model evaluation and cross validation

10-fold cross-validation tests were performed to assess the performance of our prediction model. In the 10-fold cross-validation tests, the training dataset was divided into10 subgroups with approximately equal size. At each cross-validation step, one subgroup was singled out as the test dataset to assess the performance of the classifier, while the classifier was trained using the remaining 9 subgroups. The performance of each cross-validation produced a single estimation and this procedure was repeated 10 times. To evaluate the model’s performance, four measurements were calculated, including accuracy (Ac), sensitivity (Sn), specificity (Sp), and Matthews’ correlation coefficient (MCC). The following formulae are used to calculate these measures:

$$\text{Ac}=\frac{TP+TN}{TP+TN+FP+FN}$$ (4)

$$\text{Sn}=\frac{TP}{TP+FN}$$ (5)

$$\text{Sp}=\frac{TN}{TN+FP}$$ (6)

$$\text{MCC}=\frac{TP\times TN-FP\times FN}{\sqrt{\left(TN+FN)\times \left(TP+FP\right)\times \left(TN+FP\right)\times (TN+FP\right)}}$$ (7)

Where *TP*, *FP*, *TN*, and *FN* represent the numbers of true positive, false positive, true negative and false negative, respectively. Furthermore, the receiver-operating characteristic (ROC) curve, which plots Sn against 1-Sp at different thresholds, was also employed for performance assessment. To further quantify the performance, the areas under the ROC curves (AUCs) were calculated by the pROC package in R software [50, 51].

## Results and Discussion

### Performance assessment on the training dataset

The iPUP training dataset was used to develop the pbPUP predictor. The ratio of positive to negative samples is nearly 1:12 in this dataset, which is highly imbalanced. It has been established that machine learning algorithms become computationally intractable and their accuracy is strongly affected due to the nature of the unbalanced datasets [52, 53]. To address this, many PTM site predictors employ a relatively balanced ratio of positives to negatives to train the classification models, including the prediction of pupylation sites as well [31, 54, 55]. In the current study, a 1:2 ratio of positives to negatives was used for the training dataset to develop the proposed SVM predictor.

The window size is an important factor of the prediction performance, which reflects the influence of surrounding residues on the discrimination of pupylation from non-pupylation sites. The window sizes ranging from 25 to 61were optimized based on the AUC values. For each window size, the SVM parameters were optimized through the grid search, and the corresponding AUC value was obtained from the 10-fold cross-validation test on the training set. As a result, the optimal window size of 57 (the corresponding optimal SVM parameters are *C* = 8 and *γ* = 2) was finally selected, though the performance increase with the window size ranging from 45–57 was not prominent (S1 Fig).

**Fig 2:**
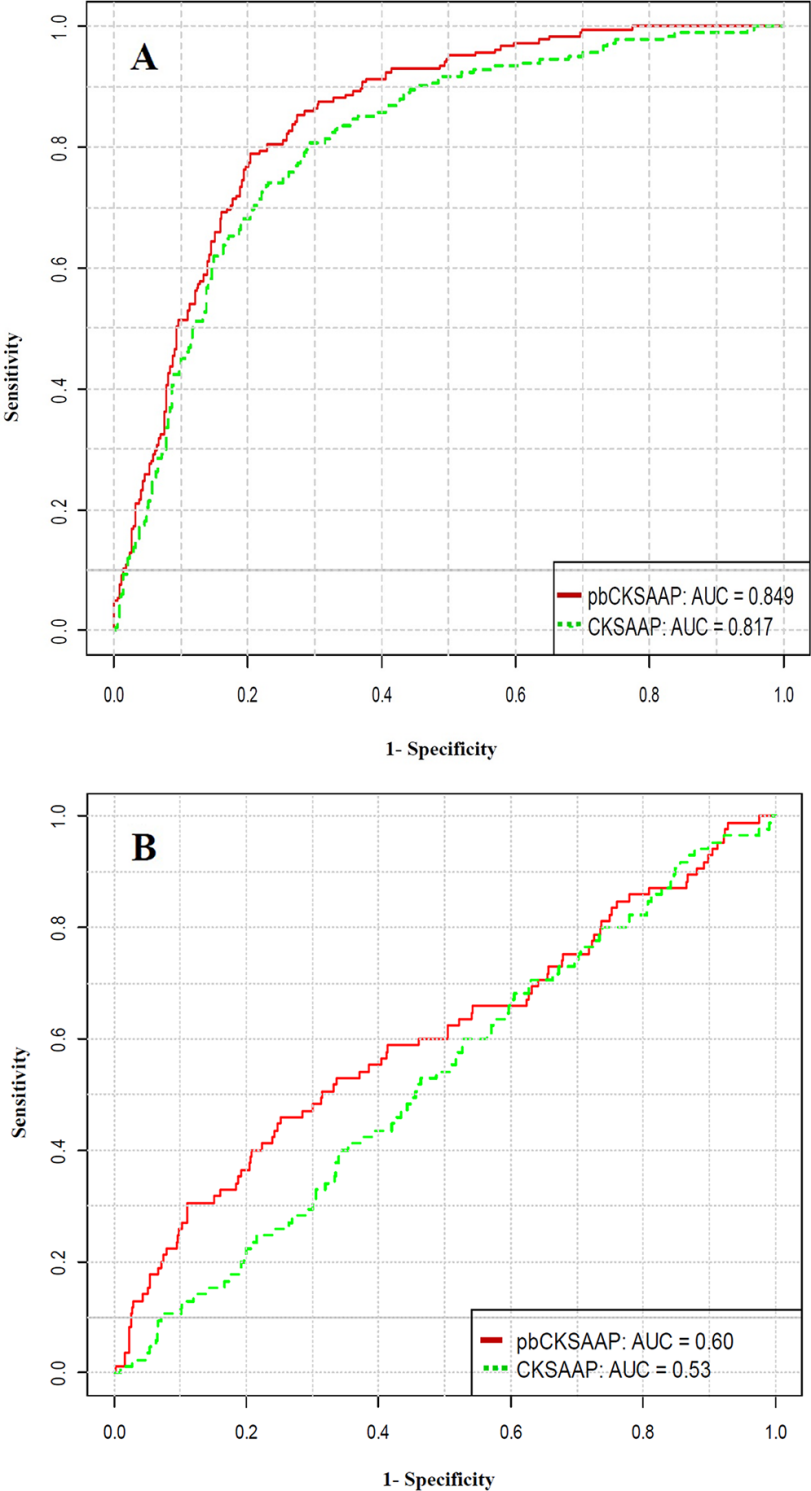
Performance comparison between pbCKSAAP and CKSAAP using ROC curves. (A) Performance comparison based on 10-fold cross-validation of the training dataset; (B) Performance comparison based on the independent test dataset.

At the 90% specificity control (SVM score≥0.0), pbPUP reached an accuracy of 76.06% (Sn = 48.15% and MCC = 0.44). Meanwhile, in terms of ROC curve (Fig 2A), pbPUP achieved an AUC value of 0.849. Furthermore, we also conducted 4-, 6-, and 8-fold cross-validation tests, and the corresponding AUC values were 0.829, 0.838 and 0.846, respectively. In summary, we conclude that pbPUP predictor provides a stable and promising performance in the cross-validation tests on the training dataset.

### Performance comparison of pbPUP with three existing predictors on the independent dataset

To compare the performance of pbPUP and three other existing predictors (iPUP, GPS-PUP, and PupPred), we compiled an independent dataset covering 71 pupylated proteins, which contain 86 pupylation and 1136 putative non-pupylation sites. Among these proteins, 20 proteins (i.e. the independent test set used in iPUP) were extracted from the original article of iPUP [29] and 51 proteins were retrieved from a recent study [25]. Although pbPUP and these three predictors did not employ the same training dataset for predicting pupylation sites, the independent dataset can allow for a generally fair performance comparison. To make the comparison, the independent data were directly submitted to the respective web servers. Note that the authors of iPUP combined the training and testing datasets when constructing the server. In other words, there were 20 proteins already included in the training data of the iPUP server. Accordingly, it is not reasonable to submit these same 20 proteins again to the iPUP server. Instead, we assessed the performance of the iPUP predictor on these 20 proteins according to their original literature. The rest 51 proteins were submitted to the iPUP server and the prediction results on the 20 proteins from the published literature were further combined for making a comparison. Similar to the other predictors, we also reported the performance of pbPUP at High, Medium and Low confidence thresholds. To make a fair comparison, the thresholds of High, Medium and Low in pbPUP were set to ensure that the corresponding specificities were controlled at the same levels as those of GPS-PUP. As shown in Table 2, the pbPUP predictor achieved an improved performance with approximately 4%, 5%, and 3% higher MCC values under high, medium, and low confidence thresholds than iPUP (Table 2). The MCCs of the pbPUP predictor were nearly 7%, 8%, and 2% higher than the GPS-PUP predictor at high, medium, and low thresholds, respectively (Table 2). In addition, the pbPUP predictor achieved MCC values of almost 9%, 5%, and 5% higher than PupPred at high, medium, and low confidence thresholds (Table 2). The performance comparison results demonstrate that our proposed pbPUP predictor provides a better or competitive performance with the other three existing predictors, indicating the encoding scheme of pbCKSAAP is very useful and powerful.

**Table 2:** The prediction performance of pbPUP and other existing predictors evaluated on the independent test dataset.

Predictor	Threshold^a^	Ac (%)	Sn (%)	Sp (%)	MCC (%)
GPS-PUP	High	83.89	19.76	88.74	6.73
	Medium	78.82	24.41	82.93	4.94
	Low	71.70	36.26	74.24	7.71
iPUP	High	81.13	29.06	84.90	9.56
	Medium	75.63	33.72	78.80	7.72
	Low	72.02	37.21	74.64	6.89
PupPred	High	88.93	9.19	94.77	4.33
	Medium	79.74	27.58	83.57	7.45
	Low	63.97	43.67	65.45	4.82
pbPUP	High	84.14	30.13	88.21	13.97
	Medium	78.72	37.65	81.79	12.46
	Low	70.15	44.70	72.05	9.38

^a^The threshold values of GPS-PUP, iPUP and PupPred were the same as those defined in the corresponding webservers. To make the performance comparison, the thresholds of High, Medium and Low in pbPUP were set as 0.06, 0.00 and -0.04, respectively. Thus, the corresponding specificities were controlled at the same levels as GPS-PUP.

**Fig 3:**
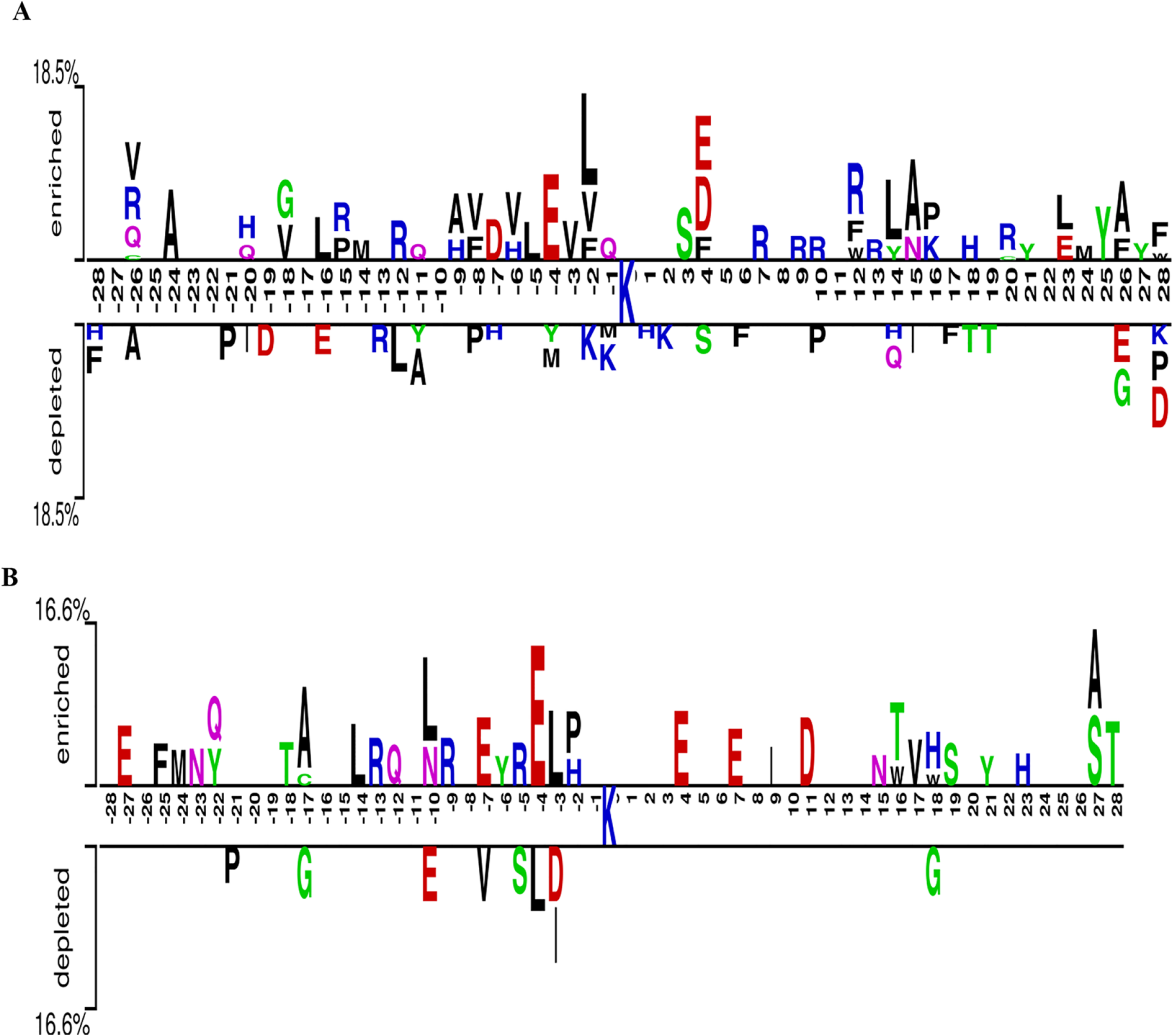
Sequence logo representations showing the amino acid occurrences between pupylation and putative non-pupylation sites. Only residues that were significantly enriched or depleted (*t*-test, *P*<0.05) flanking the centred pupylation sites are shown. Panel A represent the two-sample logo of the iPUP training dataset, while panel B plots the two-sample logo of the independent test dataset. The two-sample sequence logos were prepared using the web server http://www.twosamplelogo.org/.

Interestingly, pbPUP and the other three existing predictors showed significantly lower performance on the independent data. Our analysis suggests that the sequence patterns of pupylation sites and surrounding regions in the training and independent datasets are highly different. The position-specific amino acid occurrences for the pupylation and putative non-pupylation sites in the training and independent datasets were visualized using the Two-Sample-Logo [56] (Fig 3). Generally, the amino acid pattern around the pupylation sites is somewhat camouflaged in the independent dataset (Fig 3B), because the independent data was collected from two distinct non-pathogenic bacteria *Escherichia coli* and *Corynebacterium glutamicum* [25, 29]. The pupylation data of the latter organism has never been considered by any of the predictors. Therefore, the collected independent dataset was novel and challenging. On one hand, by intensively exploiting evolutionary information, pbPUP could achieve better performance on these novel data. On the other hand, there might exist species-specific pupylation site patterns, similar to other PTM types such as acetylation [55]. Accordingly, more comprehensive predictors (e.g. species-specific predictors) need to be developed when more pupylation data become available in the future.

### The influence of sequence redundancy on the predictive performance

The sequence redundancy might lead to the overestimation of prediction performance. Therefore, we adopted two approaches to remove the redundant sequences: 1) BLASTClust (http://www.ncbi.nlm.nih.gov/BLAST/docs/blastclust.html) was applied to remove redundant protein sequences with the 30% identity cutoff (i.e. redundancy removal at the protein level); 2) An in-house PERL script was used to remove redundant pupylated/non- pupylated peptides (also with 30% identity cutoff) at the peptide level. It is noteworthy that, as mentioned above, the authors of iPUP combined the training and testing datasets when they constructed the iPUP server. It is therefore not reasonable to submit any of the 20 proteins again to the iPUP server. To make a fair performance comparison, we had to keep these 20 proteins as they were (i.e., no redundancy removal procedure was applied to these 20 proteins), and used the performance reported in their original literature to evaluate predictors’ performance on these 20 proteins.

After removing the protein level sequence redundancy, we re-assembled a training dataset that contained 129 proteins with 149 pupylation and 298 non-pupylation sites (with the consistent 1:2 ratio of positives to negatives), and a testing dataset that contained 64 proteins with 76 pupylated and 1049 non-pupylation sites. As shown in S2 Fig, the overall performance of pbPUP in the 10-fold cross-validation decreased slightly (AUC = 0.841) after removal of the protein sequence redundancy. Furthermore, pbPUP could still achieve the best performance on the independent testing dataset (S3 Table). For example, when compared with iPUP, pbPUP achieved MCC values of approximately 4%, 3% and 1% higher under high, medium, and low thresholds, respectively. pbPUP also achieved at least a 2% MCC improvement compared with PupPred and GSP-PUP at any of the three confidence thresholds. These performance comparison results prove that pbPUP predictor provides a better or competitive performance with the other three existing predictors on the independent test datasets, even after removal of the protein sequence redundancy.

In addition, we examined the predictors’ performance after removing the peptide-level sequence redundancy. A training dataset including 148 pupylated sites and 296 non-pupylated sites were accordingly obtained (with the consistent 1:2 ratio of positives to negatives). Similar to the situation after protein-level sequence redundancy removal, there was only a small change of the overall cross-validation performance (AUC = 0.837). The independent test dataset after removal of the peptide-level sequence redundancy included 79 pupylated sites and 992 non-pupylated sites. On this dataset, pbPUP achieved the MCC values of 4%, 2%, 1% higher than iPUP at high, medium, and low confidence thresholds (S4 Table). Likewise, the MCC values of the pbPUP predictor was nearly 7%, 5%, and 1% better than the GPS-PUP predictor and 10%, 4%, and 2% better than PupPred at the corresponding thresholds (S4 Table). Altogether, we conclude that pbPUP predictor achieves a stable and competitive performance compared with other methods under both sequence-level and peptide-level sequence redundancy reduction conditions.

### Comparison of the pbCKSAAP and CKSAAP encoding schemes

The CKSAAP encoding has been previously used for prediction of pupylation sites (i.e. the iPUP predictor) [29], and the aforementioned independent test has clearly shown that our pbPUP can outperform iPUP. Since the encoding schemes of pbCKSAAP and CKSAAP are developed based on a similar strategy, it is of particular interest to comprehensively compare these two encoding schemes. To this end, we re-trained the CKSAAP-based SVM model using the training dataset in this work. Note that the window size and SVM parameters were the same as those used for training pbPUP. Based on the 10-fold cross-validation tests, pbCKSAAP outperformed the conventional CKSAAP considerably (Fig 2A).The AUC value of pbCKSAAP was approximately 3% higher than that of CKSAAP. Moreover, pbCKSAAP achieved MCC, Ac, and Sn of about 4%, 2%, and 7% higher than CKSAAP, respectively, at the fixed Sp of 90%. In addition, on the independent test dataset, the pbCKSAAP method also achieved an AUC value of approximately 7% higher than CKSAAP for pupylation site prediction (Fig 2B). These results again suggest that pbCKSAAP achieved a significant performance improvement over CKSAAP for predicting pupylation sites.

**Fig 4:**
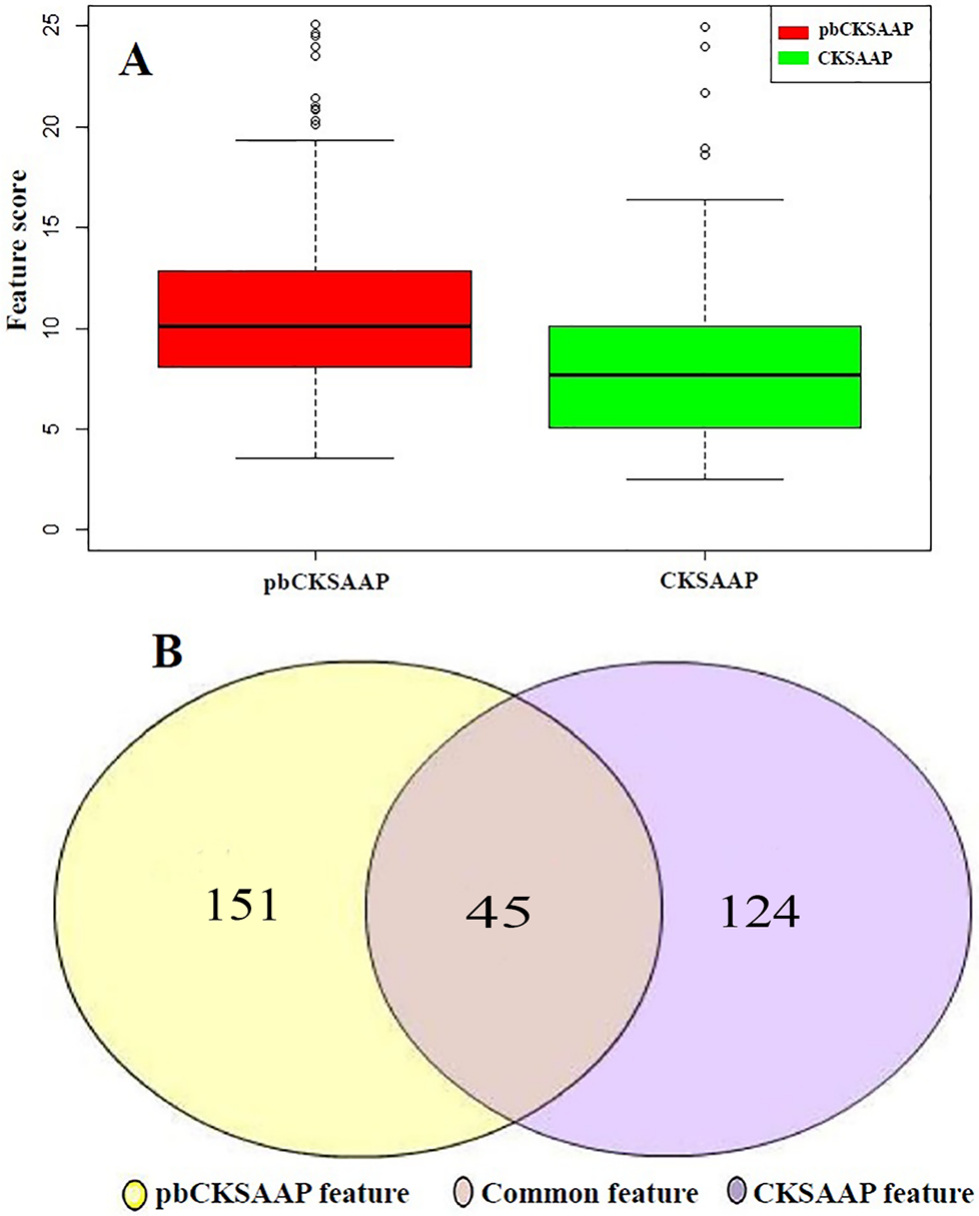
Comparison of the selected features in pbCKSAAP and CKSAAP using the *χ*² feature selection method. (A) Feature scores of pbCKSAAP and CKSAAP; (B) The numbers of selected features in pbCKSAAP and CKSAAP with the same feature selection score cutoff *χ*²≥3.

To further compare pbCKSAAP with CKSAAP, the *χ*² feature selection method was applied to select the most important pbCKSAAP and CKSAAP features. In particular, we found that the average *χ*² feature score of pbCKSAAP features was much higher than that of CKSAAP features (Fig 4A). This suggests that the pbCKSAAP features contained more important information than the CKSAAP features. To make a stringent comparison, we used the same feature score cutoff (i.e. *χ*²≥3) to select more informative features from both CKSAAP and pbCKSAAP sequence encodings. When this cutoff was applied, the number of selected pbCKSAAP features was 196, while the number of selected CKSAAP features was only 169 (Fig 4B). The number of common features shared by both pbCKSAAP and CKSAAP was 45 (Fig 4B). In summary, we conclude that pbCKSAAP contained more informative features than CKSAAP, which provides an important evidence to explain the better performance of pbCKSAAP.

**Fig 5:**
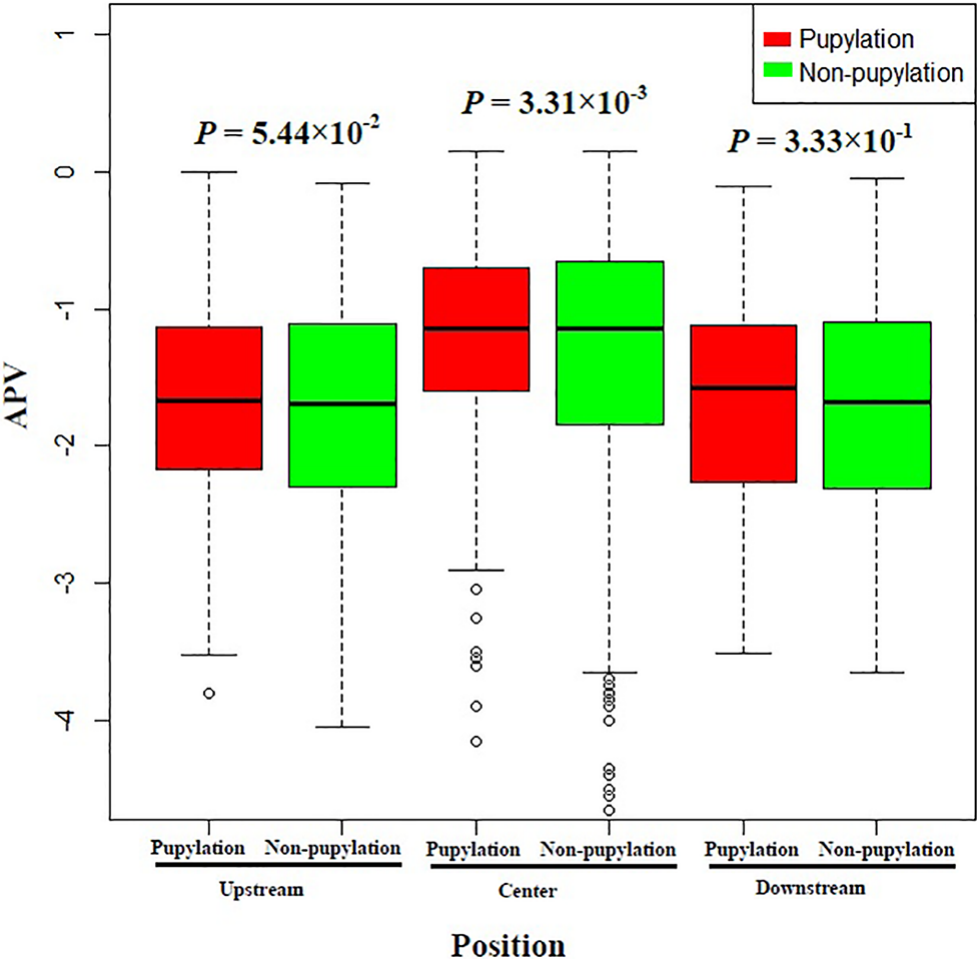
Box plots of the average PSSM values (APV) of amino acids positioned in the upstream, center, and downstream regions of pupylation and non-pupylation sites. Red color denotes pupylation sites, while green color denotes non-pupylation sites.

Compared with CKSAAP, pbCKSAAP is able to capture the evolutionary information contained in the PSSM matrix, which may explain the performance difference between CKSAAP and pbCKSAAP. In other words, the better performance of pbCKSAAP suggests that the residue conservation patterns of pupylation sites are significantly different from those of non-pupylation sites. To support our speculation, we calculated the average PSSM score (APV) of each residue surrounding pupylation and non-pupylation sites, as a useful indicator of residue conservation. The scores were calculated from each line of the PSSM matrix of the given sequences. In particular, the average PSSM values (APV) were summarized for the upstream (positions from -28 to -1), center (position 0 or central lysine) and downstream (positions from +1 to +28) regions surrounding pupylation sites. The evolutionary conservation scores of PSSM between pupylation and non-pupylation sites are illustrated in Fig 5. *P*-values were also calculated using the one-tailed *t*-test for residue positions in the upstream, center and downstream regions between pupylation and non-pupylation sequence fragments. As a result, we found that the *P*-values in the upstream and downstream regions were greater than 0.05 (*P* = 0.333 and 5.44×10^−2^, respectively), which means that the two groups of samples were not significantly different. Nevertheless, certain adjacent amino acid positions surrounding pupylation sites had significantly higher APV scores, especially the upstream positions -25, -8, -3,-4, -1 and downstream positions +3, +4, +7, +8, +11, +15, +18, +22, +25 (S3 Fig). On the other hand, *P*-value in the center region of lysine position was also less than 0.05 (*P* = 3.31×10^−3^), which suggests that pupylation sites are relatively more conserved (Fig 5). Altogether, our results confirm that the local regions surrounding pupylation sites have more conserved sequence patterns than the non-pupylation counterparts, which might possibly explain why the pbCKSAAP scheme performed better than the simple CKSAAP scheme for this prediction task.

### Significant features of pbCKSAAP

**Fig 6:**
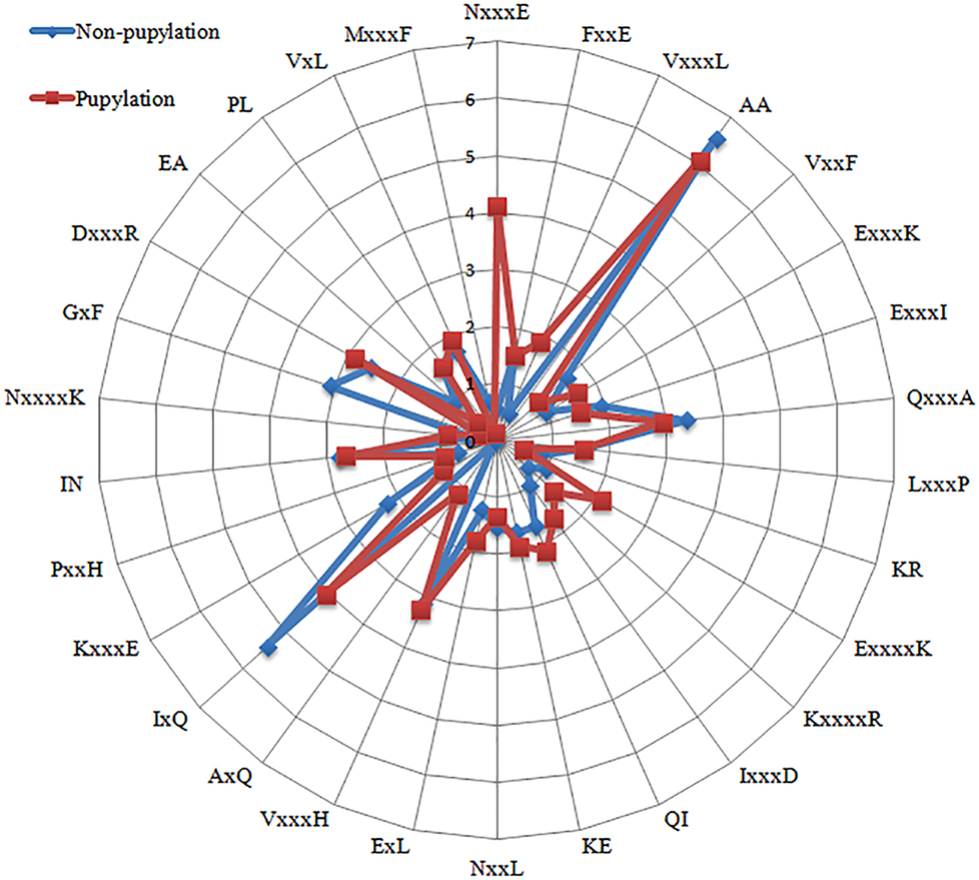
Top 30 amino acid pairs selected by the *χ*² feature selection method. Red color denotes pupylation sites, while blue color denotes non-pupylation sites. The radar diagram is represented by the composition of each residue pair whose length is proportional to the composition of pbCKSAAP features.

As mentioned above, a well-established feature selection method ***χ*²** was used to select the most important features from the high-dimensional pbCKSAAP encoding that contributed to the performance. We performed multiple rounds of experiments to select appropriate feature sets; however, it turned out that there was no significant improvement in the corresponding performance using the selected features. Probably due to the fact that SVM has a good tolerance of high-dimensional input features, the feature selection did not result in a better SVM model, which is consistent with the observations in our previous studies. Therefore, feature selection was not utilized in our final predictor. Although the feature selection strategy did not lead to significant performance improvement, we identified the top ranked 30 amino acid pairs for the purpose of investigating the most significant residues and positions surrounding pupylation and non-pupylation sites. The top 30 residue pair scores and their corresponding positions are listed in S5 Table. These important features are also presented in a radar diagram (Fig 6). The feature ‘N×××E’ (i.e. 3-spaced residue pair of ‘NE’, where ‘×’ stands for any residue) was the most important amino acid pair, representing the most enriched motif surrounding pupylation sites. Similarly, the feature ‘AA’ which represents a 0-spaced residue pair of ‘AA’ is the most important and enriched in the non-pupylated sites (Fig 6). Interestingly, the majority of the top 30 features contain charged residues such as K, R, H, E, and D (Fig 6), indicating that charged residues may play an important role in the recognition of pupylation sites. We also observed that amino acid pairs that cover all possible *k* values (i.e. *k* = 0, 1, 2, 3 and 4) were included as the most significant features (Fig 6), suggesting that all spaced amino acid pairs are necessary to make a collective contribution to the prediction of pupylation sites.

**Fig 7:**
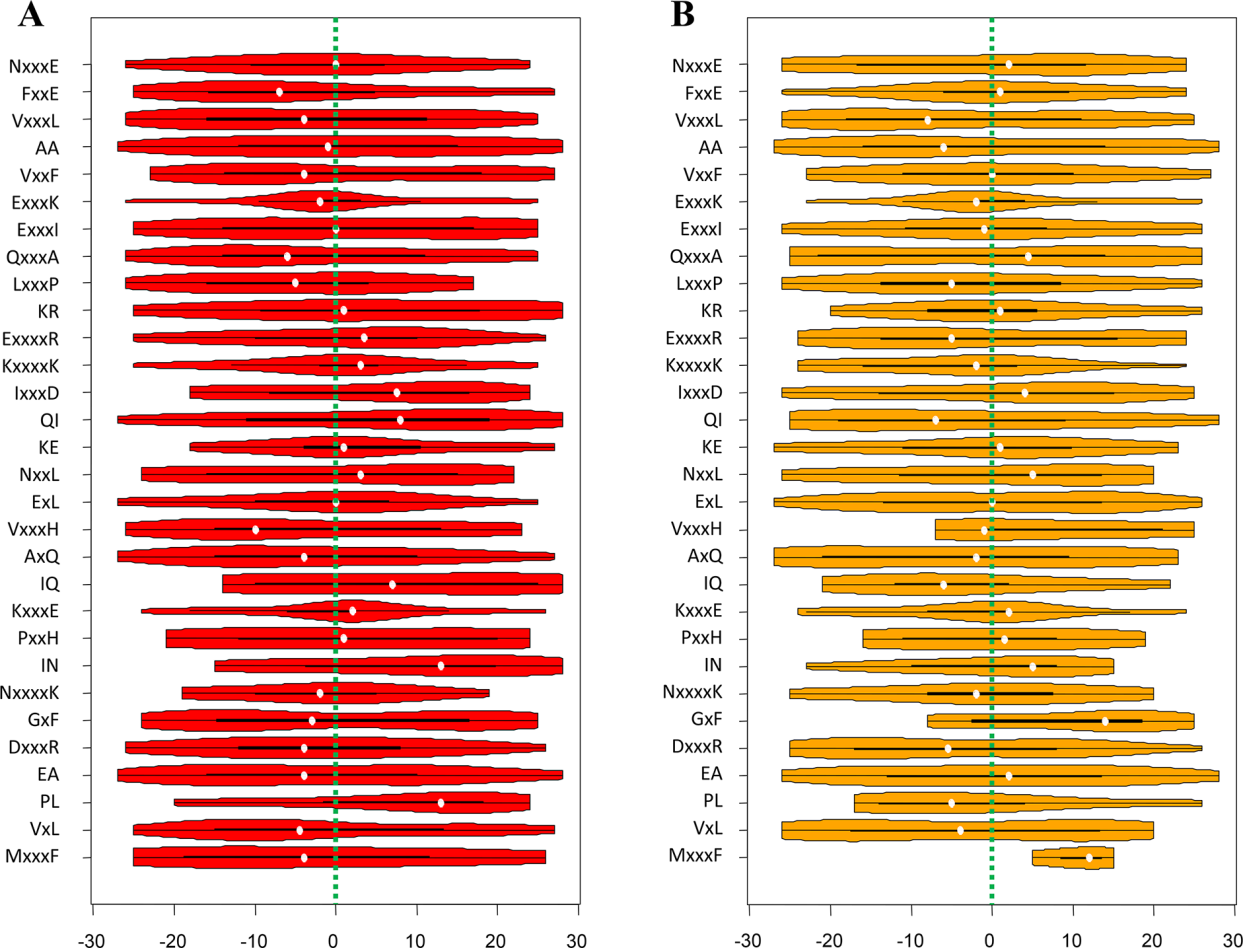
The violin plots illustrating the positional distributions of the top 30 amino acid pairs of the pbCKSAAP encoding on the pupylated peptides. (A) The distributions on the pupylated peptides from the training samples; (B) The distributions on the pupylated peptides from the independent testing samples. The white dots indicate the median values, the black boxes indicate the ranges between 1^st^ quartiles and 3^rd^ quartiles, while the outskirt violin-like shapes denote the probability destiny plots. For clarity, green dashed lines indicating the position of the central lysines are also added.

Although the SVM framework what pbPUP used is a black-box computational model, the above analyses have provided clues for interpreting the biological knowledge of the pbCKSAAP encoding scheme. That is, the pbCKSAAP encoding is able to represent and depict the weakly conserved motifs hidden in the surrounding sequences of pupylation sites. Three important properties of this encoding should be highlighted. The first one is the usage of *k*-spaced amino acid pair. As a sketch of sequence motif, *k*-spaced amino acid pair could better reflect the coordinated pairs of residues surrounding the pupylation sites. Indeed, as shown in Fig 6 and S5 Table, amino acid pairs covering all possible *k* values (i.e. *k* = 0, 1, 2, 3 and 4) and almost all of the 20 amino acids (except rare amino acids like C, W, Y) could be found in the list of the top 30 most-informative features. These results indicate that the spectrum of possible *k*-spaced amino acid pairs could serve as an enriched and explicit representation of the sequence patterns. The second key property of the pbCKSAAP encoding is the usage of position-independent composition encoding. We mapped the top 30 informative amino acid pairs onto the pupylated peptides in both the training and testing datasets, respectively (Fig 7). It is obvious that most of them did not exhibit concentrated distributions, but were instead dispersed along the peptide fragment. Even for the amino acid pairs that showed obvious concentrated distributions (e.g. FxxE and KxxxxK), their distributions were still somehow different in the training and testing samples (Fig 7). For instance, the distribution of FxxE shifted towards the downstream in the testing samples, while the distribution of KxxxxK shifted towards the upstream in the training samples. Therefore, in this situation, the position-independent encoding might be able to better describe the sequence patterns than a position-dependent encoding. On the other hand, it is also noticeable that pbCKSAAP did not completely disregard other informative position-dependent amino acid patterns. For example, a conserved enrichment of E at positions -4 and +4 was observed in the flanking sequences of pupylation sites (Fig 3). Accordingly, the amino acid pairs ExxxK and KxxxE were ranked among the top features (Fig 6) and exhibited conserved positional distributions in the training and testing samples (Fig 7). Last but not least, pbCKSAAP embedded the evolutionary information into its encoding. Our previous analysis has shown that several positions flanking the pupylation sites were slightly more conserved than the corresponding positions of non-pupylation sites (S3 Fig). pbCKSAAP took advantage of this weak conservation pattern to prioritize the weakly conserved amino acid pairs. To characterize the pbCKSAAP-specific features and CKSAAP-specific features (Fig 4B), we compared the numbers of their matched pupylated peptides on the independent testing dataset. As shown in S4 Fig, pbCKSAAP-specific features generally matched more pupylated peptides than CKSAAP-specific features. Especially, the fraction of zero-matched features of pbCKSAAP-specific features was significantly smaller than that of CKSAAP-specific features, indicating that pbCKSAAP is able to extract weakly conserved amino acid pairs to achieve more accurate prediction performance.

### Web server implementation

As an implementation of our method, a web server of pbPUP (profile-based pupylation site predictor) has been made available at http://protein.cau.edu.cn/pbPUP/ to the research community. The web server was developed using Perl, CGI scripts, PHP and HTML. The input and exemplar output web pages of the server are shown in S5A and S5B Fig, respectively. In the input web page, users can submit their query sequence by pasting it into the text box. After submitting the query sequence to the server, it will initially generate lysine fragments of all candidate pupylation sites. Simultaneously, the server will generate the PSSM matrix of the query sequence by performing PSI-BLAST search and calculate the pbCKSAAP encodings for all the generated fragments. Finally, the server will calculate the prediction scores of all the fragments with the assistance of SVM classifier. After the submission job is completed, the server will return the prediction result in the output webpage, consisting of the job ID, the query protein name, residue position, and the SVM score of the predicted pupylation sites in a tabular form. Note that the current pbPUP server predicts pupylation sites at the 90% specificity control. Users can also view the results in the text format. The prediction results will be generated for all candidate lysine residues of the submitted sequence. User will receive a job ID and can save this ID for future query. Our server stores this job ID for one month.

## Conclusion

In this study, we have developed an efficient approach termed as pbPUP for improving the prediction of protein pupylation sites. Benchmarking experiments based on cross-validation and independent tests have shown that pbPUP provides a competitive performance compared with several existing methods. We have also shown that the proposed sequence encoding scheme pbCKSAAP outperformed the conventional CKSAAP encoding scheme. Our analysis suggests that the pbCKSAAP encoding is able to capture important sequence evolutionary information, which plays an important role for the performance improvement. Moreover, we performed feature selection experiments to characterize the contributive features and facilitate better understanding and interpretation of our prediction model. Computational analyses also demonstrate that our proposed method can be used as a powerful tool for understanding the mechanism of protein pupylation. Finally, we have also implemented a user-friendly web server for the research community, which is freely available at http://protein.cau.edu.cn/pbPUP/.

## Supporting Information

S1 Table List of training data. (XLSX)

S2 Table List of independent data. (XLSX)

S3 Table The prediction performance of pbPUP and other existing predictors on the independent test dataset after the removal of protein-level sequence redundancy. (DOC)

S4 Table The prediction performance of pbPUP and other existing predictors on the independent test dataset after the removal of peptide-level sequence redundancy. (DOC)

S5 Table The most important features and their corresponding feature selection scores. (DOCX)

S1 Fig AUC values for different window sizes based on 10-fold cross-validation tests. (DOCX)

S2 Fig ROC curves after the application of different sequence redundancy removal methods (at either protein- or peptide-level), according to 10-fold cross-validation tests. (DOCX)

S3 Fig Average PSSM values (APV) at different positions of positive and negative fragments. P-values were calculated using the one-tailed t-test. *, *P*<0.01.
(DOCX)

S4 Fig The distribution of matched pupylated peptides of the selected amino acid pair features in the testing dataset. The matched pupylated peptides of pbCKSAAP-specific features and CKSAAP-specific features were considered, respectively.
(DOCX)

S5 Fig (A) The input page of the pbPUP server. Users can paste the query sequence into the text box and submit the prediction job; (B) The output page of the pbPUP server, which provides an example output of the prediction result for the query sequence.
(DOCX)
