# Supplementary material for: Computational Identification of Protein Pupylation Sites by Using Profile-Based Composition of k-Spaced Amino Acid Pairs
Source: PLoS One. 2015 Jun 16;10(6):e0129635. doi: 10.1371/journal.pone.0129635 (PMC4469302; doi:10.1371/journal.pone.0129635)
Supplement: S3 Table — (DOC) [file pone.0129635.s003.doc]

**Table S3**. The prediction performance of pbPUP and other existing predictors on the independent test dataset after the removal of protein-level sequence redundancy.

| Predictor | Threshold^a^ | Ac (%) | Sn (%) | Sp (%) | MCC (%) |
| --- | --- | --- | --- | --- | --- |
| GPS-PUP | High | 84.00 | 19.73 | 88.65 | 6.50 |
|  | Medium | 78.48 | 23.68 | 82.46 | 4.02 |
|  | Low | 70.49 | 36.84 | 72.93 | 5.48 |
| iPUP | High | 80.42 | 28.94 | 84.16 | 8.81 |
|  | Medium | 75.37 | 32.89 | 78.45 | 6.84 |
|  | Low | 71.53 | 36.84 | 74.04 | 6.18 |
| PupPred | High | 88.82 | 10.14 | 94.07 | 4.26 |
|  | Medium | 79.29 | 25.33 | 83.23 | 5.70 |
|  | Low | 63.13 | 42.67 | 64.61 | 3.82 |
| pbPUP | High | 84.36 | 27.27 | 88.56 | 12.67 |
|  | Medium | 78.76 | 34.46 | 81.97 | 9.46 |
|  | Low | 71.11 | 41.53 | 73.25 | 7.48 |

^a^ The thresholds were taken as described in Table 2.
