# Supplementary material for: Computational Identification of Protein Pupylation Sites by Using Profile-Based Composition of k-Spaced Amino Acid Pairs
Source: PLoS One. 2015 Jun 16;10(6):e0129635. doi: 10.1371/journal.pone.0129635 (PMC4469302; doi:10.1371/journal.pone.0129635)
Supplement: S4 Table — (DOC) [file pone.0129635.s004.doc]

**Table S4**. The prediction performance of pbPUP and other existing predictors on the independent test dataset after removal of peptide-level sequence redundancy.

| Predictor | Threshold^a^ | Ac (%) | Sn (%) | Sp (%) | MCC (%) |
| --- | --- | --- | --- | --- | --- |
| GPS-PUP | High | 84.31 | 16.66 | 89.62 | 5.26 |
|  | Medium | 79.27 | 21.79 | 83.79 | 3.89 |
|  | Low | 71.98 | 35.89 | 74.82 | 6.35 |
| iPUP | High | 79.72 | 28.21 | 83.77 | 8.27 |
|  | Medium | 75.24 | 32.05 | 78.46 | 6.41 |
|  | Low | 70.74 | 37.17 | 73.38 | 6.16 |
| PupPred | High | 88.23 | 7.69 | 94.56 | 2.55 |
|  | Medium | 81.41 | 20.51 | 86.20 | 4.98 |
|  | Low | 65.82 | 42.30 | 67.67 | 5.51 |
| pbPUP | High | 84.79 | 25.51 | 89.61 | 12.35 |
|  | Medium | 79.45 | 28.50 | 83.53 | 8.66 |
|  | Low | 73.08 | 36.44 | 76.01 | 7.27 |

^a^ The thresholds were taken as described in Table 2.
