# Supplementary material for: Computational Identification of Protein Pupylation Sites by Using Profile-Based Composition of k-Spaced Amino Acid Pairs
Source: PLoS One. 2015 Jun 16;10(6):e0129635. doi: 10.1371/journal.pone.0129635 (PMC4469302; doi:10.1371/journal.pone.0129635)
Supplement: S5 Table — (DOCX) [file pone.0129635.s005.docx]

**Table S5.** The most important features and their corresponding feature selection scores.

| Top 30 ranks | Score | Pair |
| --- | --- | --- |
| 1 | 32.85 | N××E |
| 2 | 26.64 | F××E |
| 3 | 25.07 | V×××L |
| 4 | 24.61 | AA |
| 5 | 24.54 | V××F |
| 6 | 23.97 | E×××K |
| 7 | 23.52 | E×××I |
| 8 | 21.46 | Q×××A |
| 9 | 21.03 | L×××P |
| 10 | 20.88 | KR |
| 11 | 20.84 | E××××K |
| 12 | 20.84 | K××××R |
| 13 | 20.35 | I×××D |
| 14 | 20.15 | QI |
| 15 | 19.36 | KE |
| 16 | 19.26 | N××L |
| 17 | 18.54 | E×L |
| 18 | 18.3 | V×××H |
| 19 | 18.3 | A×Q |
| 20 | 18.19 | I×Q |
| 21 | 17.96 | K×××E |
| 22 | 17.74 | P××H |
| 23 | 17.34 | IN |
| 24  25  26  27  28  29  30 | 17.04  16.75  16.75  16.21  15.72  15.71  15.11 | N××××K  G×F  D×××R  EA  PL  V×L  M×××F |

For example, the feature 'NxxE' represents a 2-spaced residue (any amino acid) pair of 'NE', where x stands for any amino acid. The same representation was applied to other k-spaced residue pairs.
