# Supplementary figures and images for: Computational Identification of Protein Pupylation Sites by Using Profile-Based Composition of k-Spaced Amino Acid Pairs
Source: PLoS One. 2015 Jun 16;10(6):e0129635. doi: 10.1371/journal.pone.0129635 (PMC4469302; doi:10.1371/journal.pone.0129635)

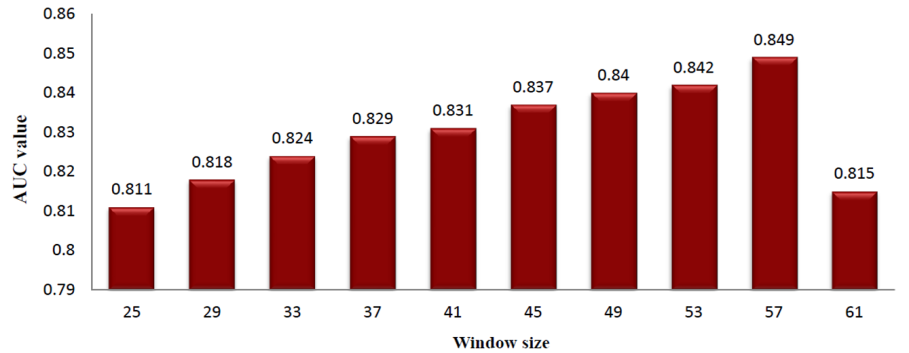


**Figure S1.** AUC values for different window sizes based on 10-fold cross-validation tests.

Supplement: S1 Fig — (DOCX) [file pone.0129635.s006.docx]
