# Supplementary material for: Computational Identification of Protein Pupylation Sites by Using Profile-Based Composition of k-Spaced Amino Acid Pairs
Source: PLoS One. 2015 Jun 16;10(6):e0129635. doi: 10.1371/journal.pone.0129635 (PMC4469302; doi:10.1371/journal.pone.0129635)
Supplement: S2 Fig — (DOCX) [file pone.0129635.s007.docx]

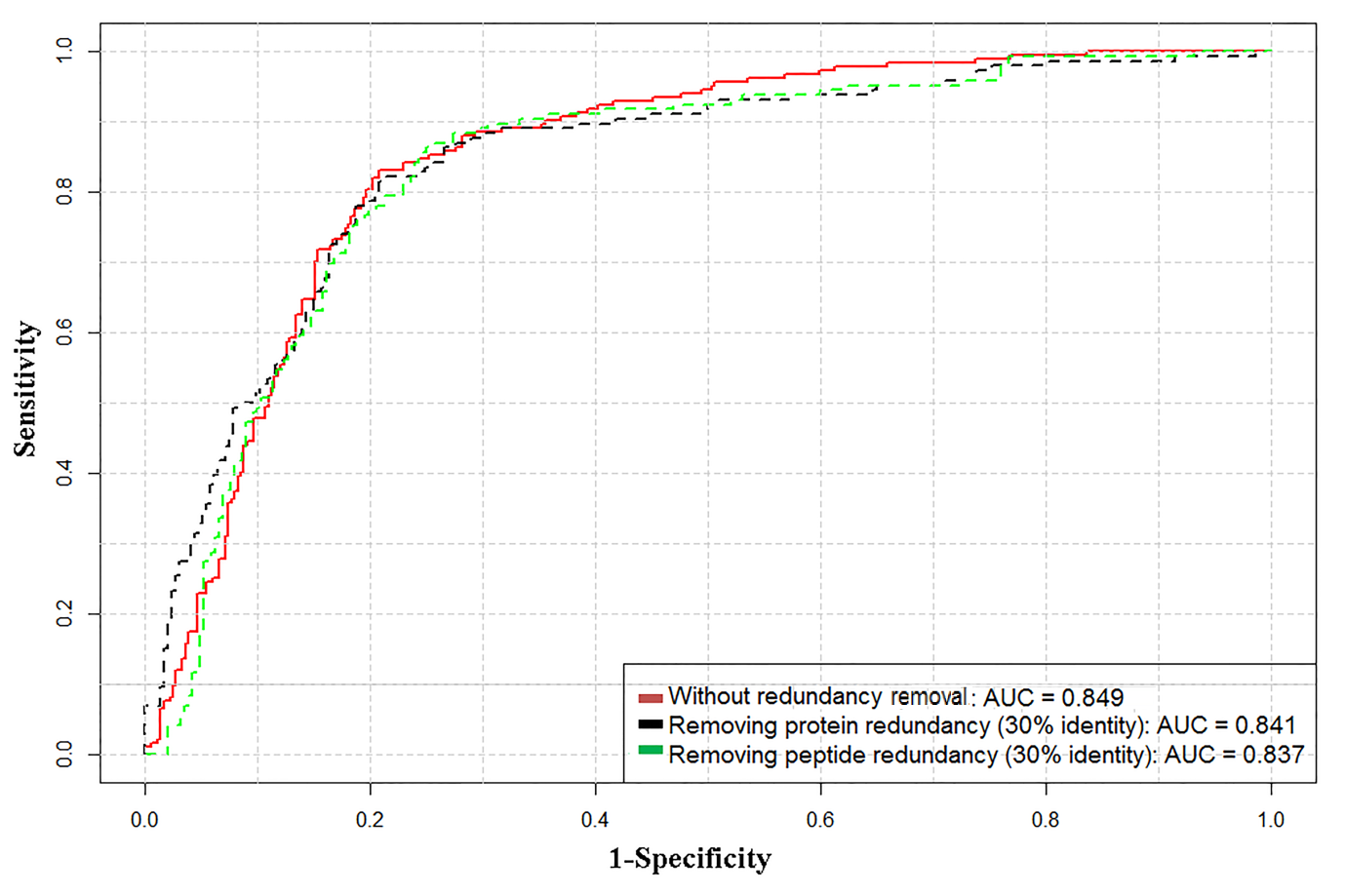


**Figure S2.** ROC curves after the application of different sequence redundancy removal methods (at either protein- or peptide-level), according to 10-fold cross-validation tests.
