# Supplementary material for: Computational Identification of Protein Pupylation Sites by Using Profile-Based Composition of k-Spaced Amino Acid Pairs
Source: PLoS One. 2015 Jun 16;10(6):e0129635. doi: 10.1371/journal.pone.0129635 (PMC4469302; doi:10.1371/journal.pone.0129635)
Supplement: S3 Fig — P-values were calculated using the one-tailed t-test. *, P<0.01. (DOCX) [file pone.0129635.s008.docx]

**
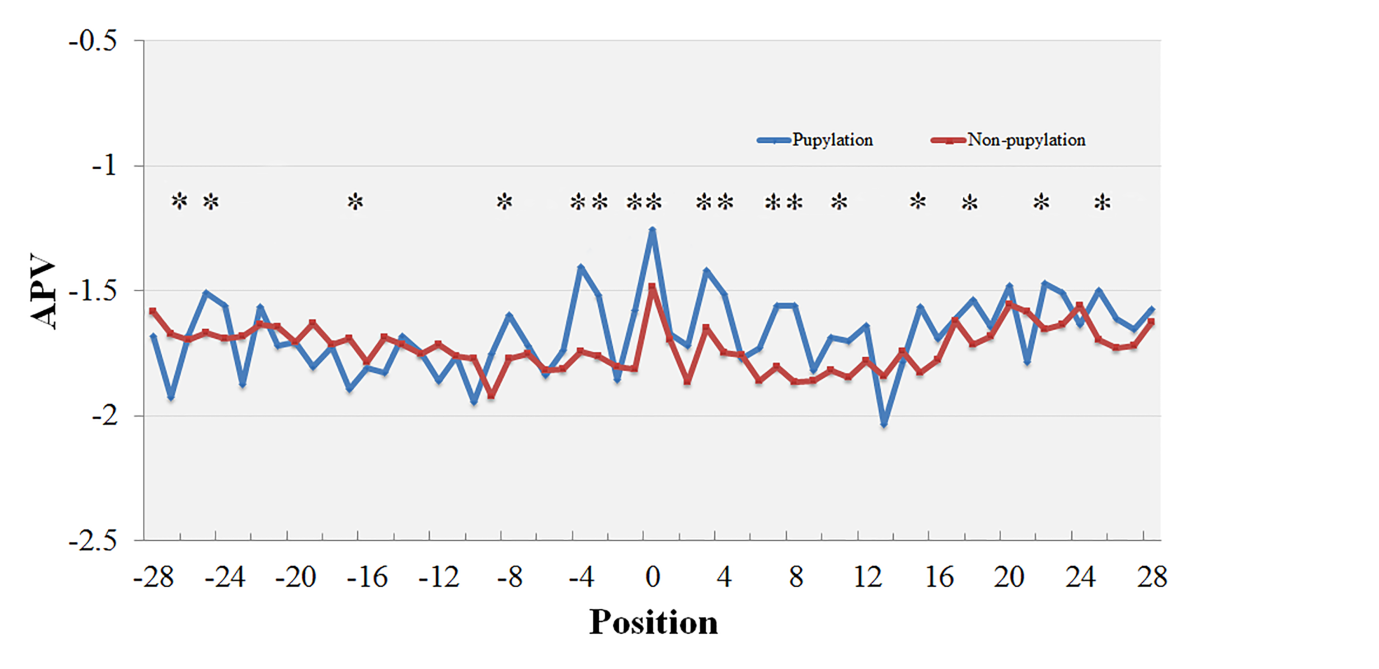
Figure S3**. Average PSSM values (APV) at different positions of positive and negative fragments. P-values were calculated using the one-tailed t-test. *, *P*<0.01.
