# Supplementary material for: Computational Identification of Protein Pupylation Sites by Using Profile-Based Composition of k-Spaced Amino Acid Pairs
Source: PLoS One. 2015 Jun 16;10(6):e0129635. doi: 10.1371/journal.pone.0129635 (PMC4469302; doi:10.1371/journal.pone.0129635)
Supplement: S4 Fig — The matched pupylated peptides of pbCKSAAP-specific features and CKSAAP-specific features were considered, respectively. (DOCX) [file pone.0129635.s009.docx]

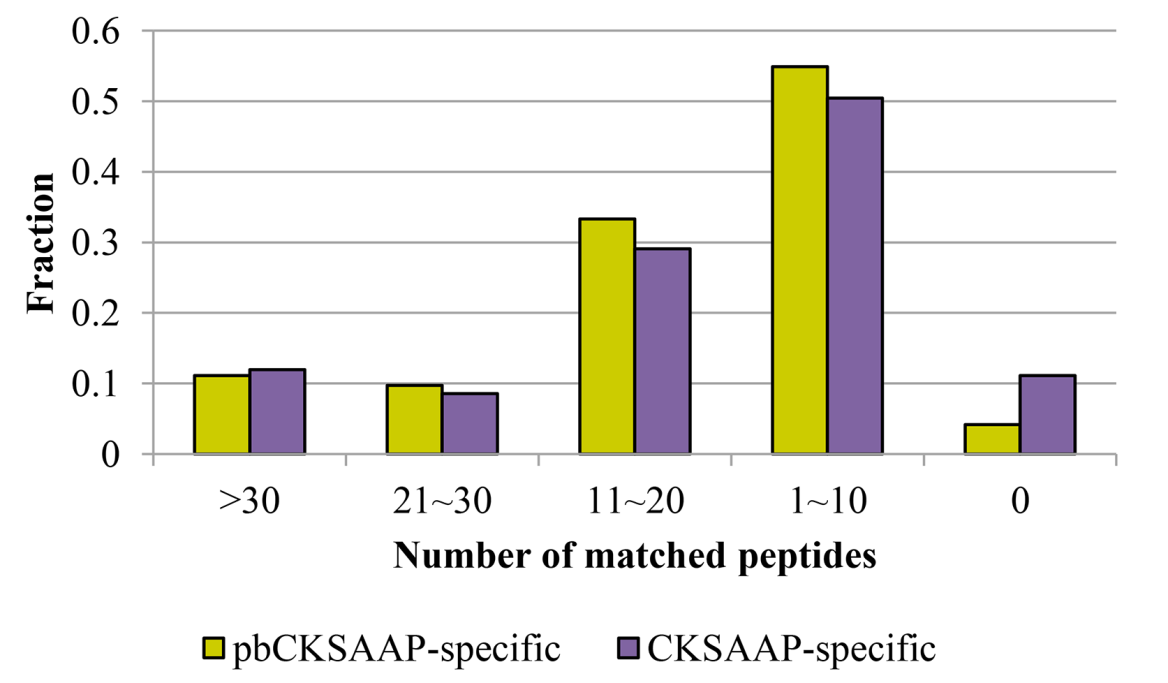


**Figure S4.** The distribution of matched pupylated peptides of the selected amino acid pair features in the testing dataset. The matched pupylated peptides of pbCKSAAP-specific features and CKSAAP-specific features were considered, respectively.
