# Supplementary material for: Computational Identification of Protein Pupylation Sites by Using Profile-Based Composition of k-Spaced Amino Acid Pairs
Source: PLoS One. 2015 Jun 16;10(6):e0129635. doi: 10.1371/journal.pone.0129635 (PMC4469302; doi:10.1371/journal.pone.0129635)
Supplement: S5 Fig — (A) The input page of the pbPUP server. Users can paste the query sequence into the text box and submit the prediction job; (B) The output page of the pbPUP server, which provides an example output of the prediction result for the query sequence. (DOCX) [file pone.0129635.s010.docx]

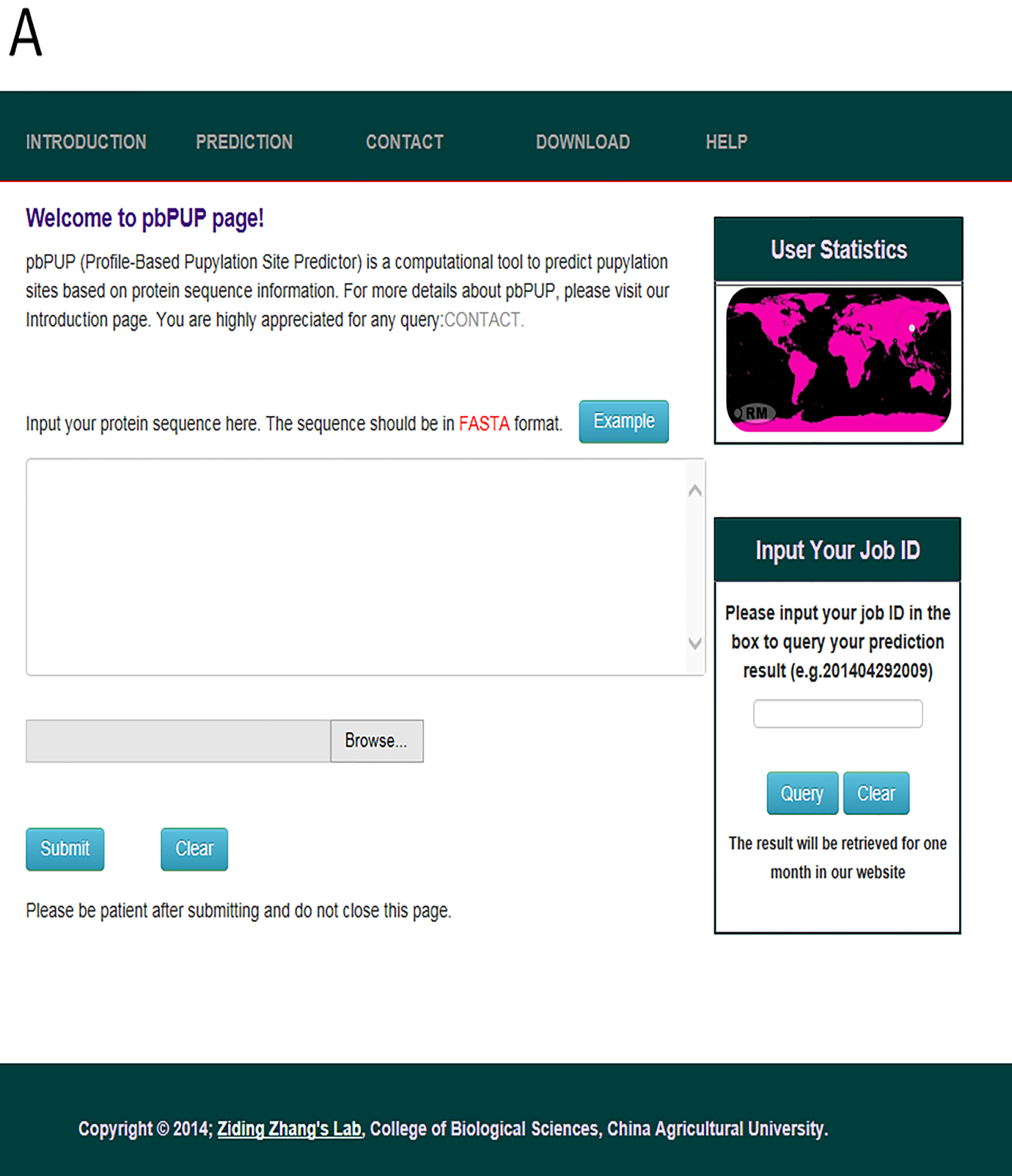


**
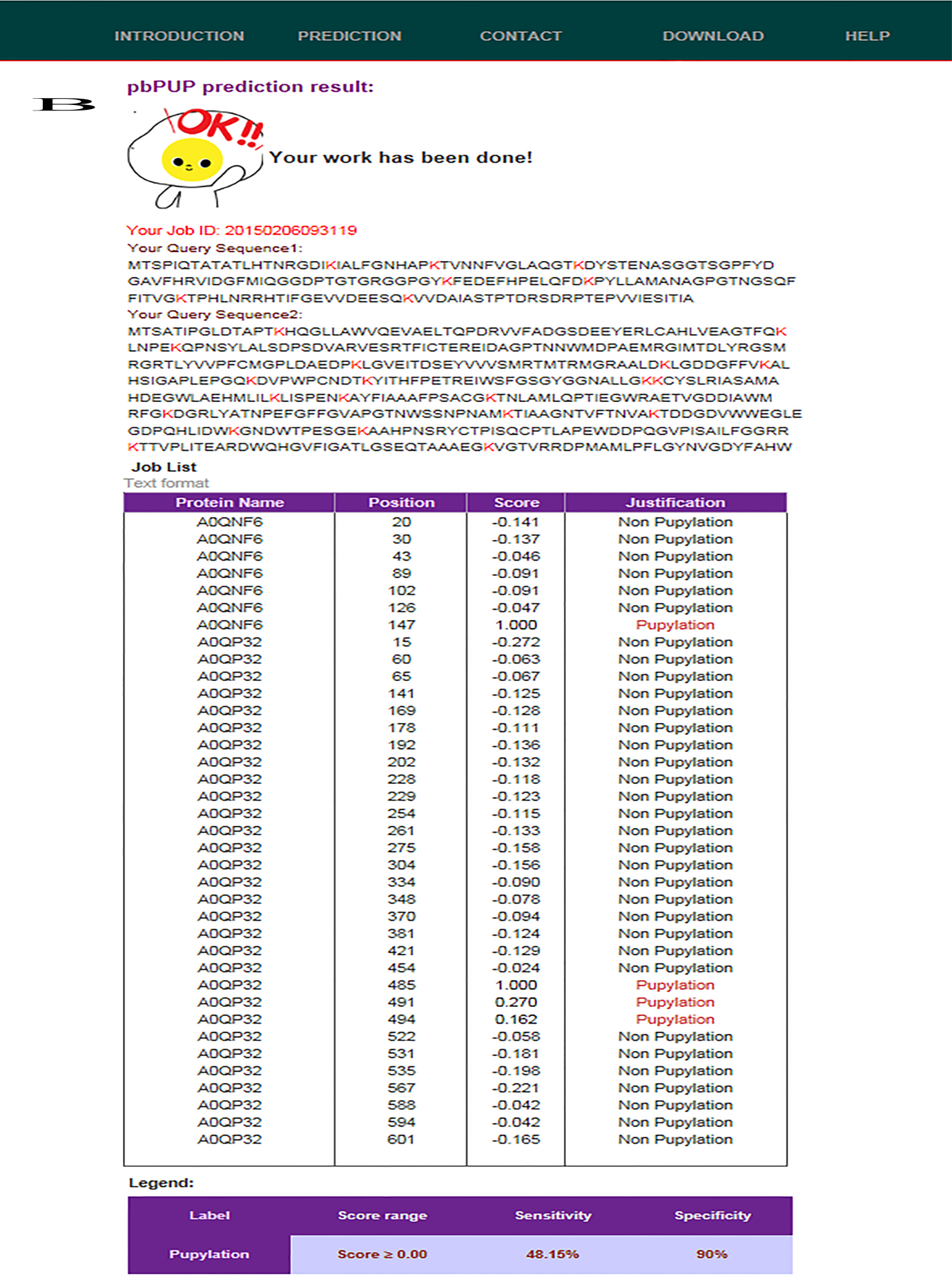
**

**Figure S5**. (A) The input page of the pbPUP server. Users can paste the query sequence into the text box and submit the prediction job; (B) The output page of the pbPUP server, which provides an example output of the prediction result for the query sequence.
